# Saltwater nectotizing fasciitis following coral reef laceration possibly exacerbated by a long-haul flight: a case report

**DOI:** 10.1186/1757-1626-2-9102

**Published:** 2009-11-27

**Authors:** Ann-Maria Byrne, Paul Sullivan, Peter Keogh

**Affiliations:** 1 Department of Orthopaedics, Connolly Hospital, Blanchardstown, Dublin 15, Ireland; 2 Department of Plastic Surgery, Connolly Hospital, Blanchardstown, Dublin 15, Ireland

## Abstract

**Introduction:**

Necrotising fasciits is a rapidly progressive disease characterised by extensive necrosis of the fascia, skin, and subcutaneous tissue, with relative sparing of the underlying muscle.

**Case presentation:**

We present the case of a 24-year old Irish male student who sustained a laceration to his right shin from contact with a coral reef while swimming in the Phuket region, off the west coast of Thailand. The following day, he returned to Ireland and presented with an aggressive and destructive variant of group A beta-hemolytic streptococcal necrotising fasciitis originating at the site of the coral reef injury, and exacerbated by the long-haul flight. He was successfully treated with aggressive surgical debridement, vacuum-assisted dressings, split skin grafting and broad spectrum antibiotics.

**Conclusion:**

Necrotising fasciitis can progress rapidly to systemic toxicity and even death without expedient diagnosis and aggressive treatment. Long-haul flights induce significant fluid accumulation in the lower extremity. These physiological fluid shifts may have contributed to the severity of our patient's necrotizing condition following his flight from Thailand.

## Introduction

Necrotising fasciits is a rapidly progressive disease characterised by extensive necrosis of the fascia, skin, and subcutaneous tissue, with relative sparing of the underlying muscle. Wilson [1], in 1952, first coined the term necrotising fasciitis to describe a severe superficial tissue infection which on histology reveals necrosis of the fascia and subcutaneous tissue. The condition can progress rapidly to systemic toxicity and even death without expedient diagnosis and aggressive treatment. Patients present with signs of progressive inflammation such as pain, erythema, swelling at the affected site [2]. Disproportionate pain levels compared with clinical findings in association with systemic toxicity should raise the suspicion of necrotising fasciitis. As infection progresses, the skin becomes increasingly tense and erythematous, changing colour sequentially from red to a dusky purple before progressing to necrosis and formation of haemorrhagic bullae [2]. Crepitus of the affected area with air in the soft tissues on plain radiography has been reported in 37% and 57% of patients, respectively [3].

Initial symptoms may be non-specific and develop insidiously over a period of hours to several days, causing the diagnosis to be delayed until the patient decompensates with toxic shock multi-organ failure. Thus, a high index of suspicion must be maintained with cases of severe and advancing cellulitis. It is recommended that management should consist of immediate resuscitation, early surgical debridement, and administration of broad spectrum intravenous antibiotics. We present the case of a young man with no previous comorbidities, who presented with necrotising fasciitis of the right leg following a coral reef injury and a long-haul flight, and was successfully treated with aggressive surgical debridement, vacuum-assisted dressings and broad spectrum antibiotics.

## Case presentation

A 24-year old Caucasian Irish male student sustained a laceration to his right shin from contact with a coral reef while swimming in the Phuket region, off the west coast of Thailand. He had spent three weeks travelling in Thailand and the day following his injury was to return home to Ireland. On the ten hour flight home, he developed symptoms of general malaise, nausea, and chills. The right shin wound had swollen dramatically with associated swelling of his right leg, and dusky lesions around his pretibial wound and the dorsum of his right foot. He could not weight-bear on his right leg and the region around his wound was exquisitely tender.

**Figure 1:**
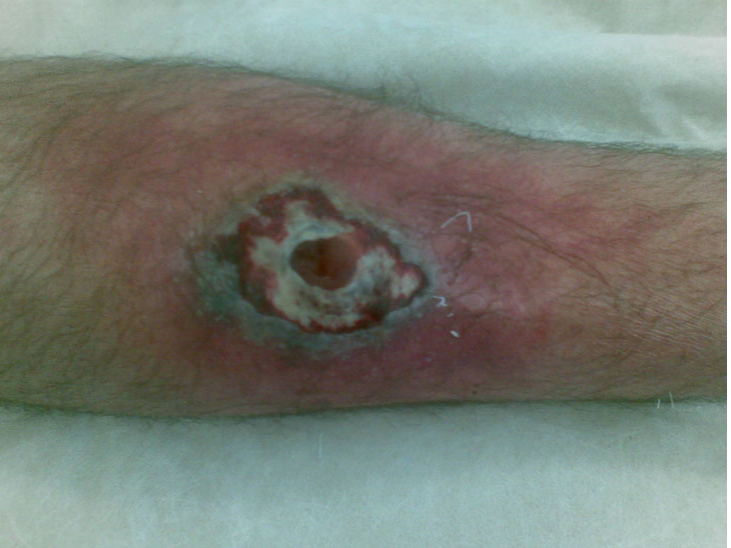
**Clinical photograph of right leg showing a 3 × 4 cm dusky area of necrotic skin over the anterior tibia with a central defect extending to the pretibial periosteum**. The entire area was surrounded by a rim of grey skin and a larger area of erythema extended down to the dorsum of the foot.

On presentation to the emergency department, a purple-black discolouration developed rapidly around the pretibial wound with skip lesions on the dorsum of the foot. A central necrotic area of skin broke down and turbid fluid extruded from the tibial wound, the anterior tibia being visible in the base of the wound. On arrival, his temperature was 38.6°C, heart rate 110 beats/min, respiratory rate 18/min, and his blood pressure was 92/60 mmHg. He was diaphoretic and clinically dehydrated. Examination of the right leg revealed a 3 × 4 cm dusky area of necrotic skin with a central punctum. The entire area was surrounded by a rim of grey skin and a larger area of erythema beyond, (Figure 1). Two further similar but much smaller areas were seen as distant skip lesions on the dorsal aspect of the foot with surrounding pitting oedema and erythema. The remaining examination was unremarkable and he had no previous medical history. Initial haematological parameters showed a white cell count of 16.6 × 10^9^/L and a neutrophil count of 14.2 × 10^9^/L. His glucose, liver and renal profiles were within normal ranges but his inflammatory markers were markedly elevated with CRP > 90 mcg/L and ESR 64 mm/hr. Blood cultures and wound swabs were sent for microbiological analysis. Plain tibial xrays did not reveal a radio-opaque foreign body within the wound. Due to his recent long-haul flight, a Doppler ultrasound was carried out to outrule a confounding deep venous thrombosis.

**Figure 2:**
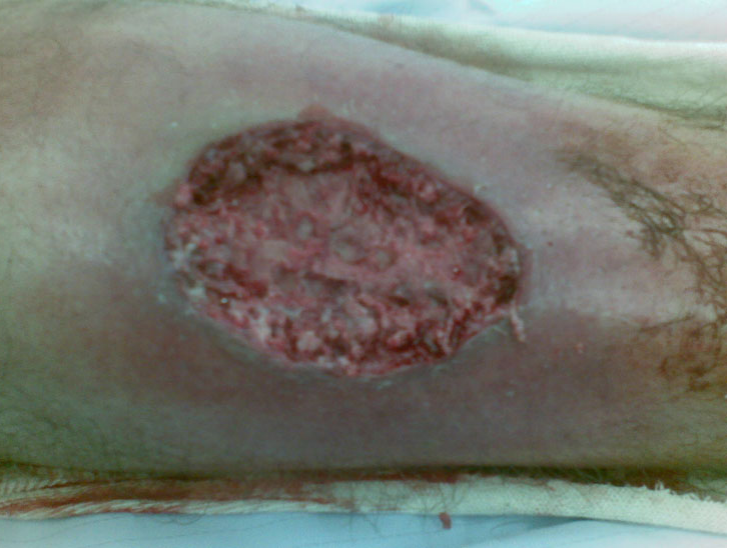
**Necrotic tissue was debrided from the right shin wound down to pretibial periosteum which was not breached**. The circumferential limits of the debridement went to bleeding skin and subcutaneous tissue.

**Figure 3:**
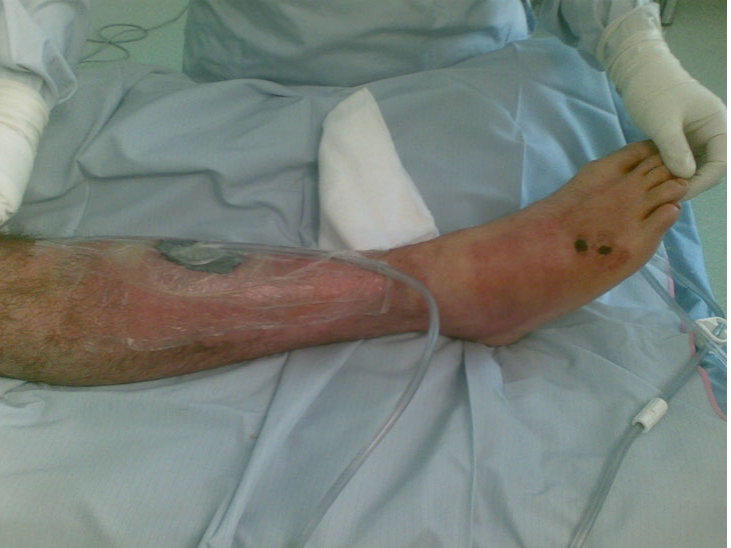
**The 7 cm × 6 cm soft tissue defect over the tibia was treated with a vacuum-assisted wound closure (VAC) device; note the presence of necrotic skip lesions on the lateral dorsum of the foot (arrowed)**.

His initial treatment involved aggressive rehydration whilst preparing for emergency surgical debridement with the suspicion of necrotising fasciitis. In the operating theatre, necrotic tissue was debrided from the shin wound down to pretibial periosteum which was not breached. The circumferential limits of the debridement went to bleeding skin and subcutaneous tissue, (Figure 2). The 7 cm × 6 cm soft tissue defect over the tibia was treated with a vacuum-assisted wound closure (VAC) device, (Figure 3) Once all microbiology and histopatholgy specimens had been taken he was started empirically on Benzylpenicillin 2.4 g and Flucloxacillin 2 g IV QDS. He twice returned to theatre for further surgical debridement at 48 hour intervals, followed by 48 to 72 hourly VAC dressing changes.

On the first post-operative day, he became pyrexial and on consultation with our microbiology colleagues, he commenced Clindamycin 900 mg IV TDS and continued on Flucloxacillin. His initial Gram stain revealed Gram-positive cocci and his definitive results identified Beta-haemolytic Group A Streptococcus as the causative organism. This was sensitive to penicillin, erythromycin and clindamycin. His histopathology specimen was reported as necrotizing fasciitis with extensive dermal lymphocytic infiltrates and widespread subepidermal oedema. With this diagnosis, he underwent thorough clinical and haematological monitoring in a high observation ward to ensure no acute decompensation requiring ICU admission.

**Figure 4:**
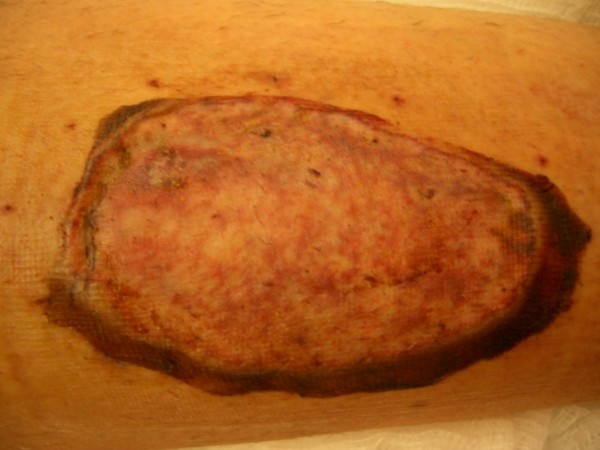
**After biochemical and inflammatory markers returned to normal, the remaining pretibial defect was covered with a split-thickness skingraft from the ipsilateral thigh**.

Plastic surgical consultation with regards to definitive wound closure was sought early in the course of treatment. After 4 weeks of IV antibiotics and VAC dressings, and when all his haematological and inflammatory markers had normalized, he returned to theatre for split skin grafting of the remaining defect (Figure 4). Following 5 days of bed rest, the graft was inspected and deemed to be a success. He commenced on a rehabilitation regime of theraband exercises to regain ankle range of motion and protected weight-bearing as tolerated. After 6 weeks in hospital he was discharged on oral antibiotics for a further week and monitored in outpatients weekly for a further 6 weeks. He went on to make a full recovery and returned to his activities of daily living without sequelae.

## Discussion

The rapidly progressive and destructive course of necrotizing fasciitis is thought to be polymicrobial rather than monomicrobial [4]. The diagnosis of primary or idiopathic necrotizing fasciitis may be challenging because it occurs in the absence of a known causative factor or portal of entry for bacteria [5]. The three main secondary necrotizing fasciitis syndromes recognized are type I, or polymicrobial; type II, or group A streptococcal; and type III gas gangrene, or clostridial myonecrosis. The organisms most closely linked to the condition are group A beta-haemolytic streptococci and this was the causative organism identified in our patient. In recent years there has been an emergence of virulent toxic shock strains of group A beta-haemolytic streptococci leading to necrotizing fasciitis with multi-organ dysfunction. Most cases are caused by M types 1 and 3 which produce exotoxin A and streptolysin O[5] The M proteins with their exotoxins act as superantigens, liberating cytokines and resulting in a toxic shock syndrome.

Another type I variant is saltwater necrotizing fasciitis, in which an apparently minor skin wound is contaminated with saltwater containing a *Vibrio *species. While our patient's coral reef injury was sustained swimming in salt water, microbiological analysis did not reveal contamination with any marine organisms. *Vibrio alginolyticus *necrotizing fasciitis has been reported in a patient following a stingray injury in the Pacific Ocean near Hong Kong [6], and in an immunocompromised patient injured on a coral reef while bathing in the Colombian coastal waters of the Caribbean Sea [7].

In assessing mortality risk in patients with necrotizing infections, Elliot et al [3], found that most patients who develop necrotising fasciitis have pre-existing conditions that render them susceptible to infection. Conditions that result in immunosuppression, such as advanced age, chronic renal failure, peripheral vascular disease, diabetes mellitus, and drug misuse appear to be risk factors. Specific conditions making patients susceptible to Vibrio infections include alcoholism and cirrhosis, oral steroid therapy, polycystic kidney disease and leukopenia, hemochromatosis, and multiple myeloma [5]. It has also been found that the condition occurs slightly more often in male patients [3]. Our patient was healthy with no associated comorbidities prior to this event, which, along with prompt diagnosis and aggressive treatment, may account for lack of critical multi-organ decompensation with this virulent and destructive infection. However, the condition may have been exacerbated by the long-haul flight and related physiological changes on the day following his coral reef injury. Mittermayr et al[8] demonstrated that long-haul flights induce significant fluid accumulation in the lower extremity and the increase in tissue oedema was maintained for some days after flight. They went on to report that lower leg volumes were significantly increased after 4 h sitting (+109 ml) reaching its maximum after 10 h (+145 ml). These changes were accompanied by an increased body weight, total body water, extracellular water and tissue thickness anterior to the tibia[8] These physiological fluid shifts may have contributed to the severity of our patient's necrotizing condition following his long-haul flight from Thailand.

Early recognition and aggressive treatment with extensive debridement and broad spectrum antibiotics is paramount to avoid the fulminant course of necrotizing fasciitis with very significant morbidity and mortality rates [3]. A further adjunct to extensive surgical debridement and dressings has been added with the use of vacuum-assisted wound closure (VAC). Huang et al [9]. compared VAC dressings with wet dressings in the management of open necrotizing limb wounds following debridement. While the VAC dressings were seven times more expensive than conventional dressings, they reported significant decreases in wound size, with treatment time reduced 3.7 fold in the VAC group. In our case, VAC dressings also provided coverage to the unbreached pretibial periosteum between surgical debridements, and, following split-skin grafting over the defect, encouraged granulation and capillary growth into the graft. Further new techniques are being examined such as hyperbaric oxygen therapy (HBO) treatment to increase tissue oxygenation in both healthy tissue and in the vicinity of infected tissue [10].

## Conclusion

Necrotising fasciitis can progress rapidly to systemic toxicity and ultimately death without expedient diagnosis and aggressive treatment. Long-haul flights induce significant fluid accumulation in the lower extremity. These physiological fluid shifts may have contributed to the severity of our patient's necrotizing condition following his flight from Thailand. An excellent clinical outcome was achieved by prompt recognition, treated with resuscitation and surgical debridement and early multidisciplinary involvement of microbiologists and plastic surgeons for antibiotic advice and reconstructive surgery.

## Abbreviations

/min: per minute; mmHg: millimetres of mercury; /L: per Litre; mcg/L: micrograms per Litre; mm/hr: millimetres per hour; cm: centimetre; g: grams; IV: intravenously; QDS: four times daily; TDS: three times daily.

## Consent

Written informed consent was obtained from the patient for publication of this case report and accompanying images. A copy of the written consent is available for review by the Editor-in-Chief of this journal.

## Competing interests

The authors declare that they have no competing interests.

## Authors' contributions

AMB prepared the manuscript, prepared intraoperative photographs and operated on the patient; PS (as a member of the plastic surgery service) prepared photographs and operated on the patient; PK was the supervising surgical consultant for this patient and contributed to the concept and preparation of this manuscript. All authors read and approved the final manuscript.
